# Candidate Genes Identified in Systemic Sclerosis-Related Pulmonary Arterial Hypertension Were Associated with Immunity, Inflammation, and Cytokines

**DOI:** 10.1155/2021/6651009

**Published:** 2021-02-18

**Authors:** Zhixiao Xu, Jiaxing Ruan, Lingyun Pan, Chengshui Chen

**Affiliations:** ^1^Department of Pulmonary and Critical Care Medicine, The First Affiliated Hospital of Wenzhou Medical University, Ouhai District, Wenzhou 325000, China; ^2^The Interventional Pulmonary Key Laboratory of Zhejiang Province, Wenzhou 325000, Zhejiang, China

## Abstract

**Background:**

Pulmonary complications of systemic sclerosis (SSc), including pulmonary arterial hypertension (PAH), are the leading causes of patient death. However, the precise molecular mechanisms of its etiology are unclear. This study's objective was to identify the candidate genes involved in the progression of SSc-PAH and investigate the genes' function.

**Methods:**

The gene expression profiles of GSE33463 were obtained from the Gene Expression Omnibus (GEO) database. A free-scale gene coexpression network was constructed using the weighted gene coexpression network analysis (WGCNA) to explore the association between gene sets and clinical features and identify candidate biomarkers. Then, gene ontology analysis was performed. A second dataset was used, GSE19617, to validate the hub genes. The verified hub genes' potential function was further explored using gene set enrichment analysis (GSEA).

**Results:**

Through average link-level clustering, a total of seven modules were classified. A total of 938 hub genes were identified in the key module, and the key module's function mainly enriched was related to chemokine activities. Subsequently, four candidate genes, BTG3, CCR2, RAB10, and TMEM60, were filtered. The expression levels of these four hub genes were consistent in the GSE19617 and GSE33463 datasets. We plotted the ROC curve of the hub genes (all AUC > 0.70). Furthermore, the results of the GSEA for hub genes were correlated with complement and inflammatory responses.

**Conclusions:**

The hub genes (BTG3, CCR2, RAB10, and TMEM60) performed well in distinguishing the SSc-PAH patients from controls, and some biological functions, related to immunity, inflammation, and cytokines, might pave the way for follow-up studies on the diagnosis and treatment of SSc-PAH.

## 1. Introduction

Systemic sclerosis (SSc), with a chronic progressive manner, is a rare systemic autoimmune disease [1]. A series of pathophysiological processes eventually lead to the deposition of excessive collagen and fibrosis in the skin and various organ systems, especially the lung [2]. Lung involvement in SSc consists of both interstitial lung diseases (ILD) and pulmonary hypertension (PAH), and it is worth noting that PAH is not uncommon in SSc [3] and might be more common in limited SSc [4]. At autopsy, PAH accounted for 8-12% of patient death [4]. It was estimated that PAH's lifetime prevalence in SSc patients is 5-12% [5].

PAH is a destructive disease that causes distinctive remodeling of the intima, medial, and adventitia layers based on the small and medium pulmonary arteries, resulting in considerable narrowing of the pulmonary vascular lumen [5–7]. The final manifestation is an increase in mean pulmonary artery pressure at rest (mPAP ≥ 25 mmHg) [5, 6]. Lung involvement is a common cause of mortality in patients with SSc [8], of which ILD is the most common, followed by PAH [6]. Moreover, it was estimated that 30% of global PAH cases appeared to be connective tissue disease-related PAH, of which SSc-related PAH (SSc-PAH) accounted for the majority [9]. The risk of death from PAH based on ILD was five times higher than that of PAH alone [10]. The prognosis of patients with SSc-PAH is abysmal. It has been reported that the 3-year mortality rate after PAH diagnosis was approximately 50% [5], while the latest 3-year survival rate was 75% [11]. In treatment, although the exercise capacity and quality of life have significantly improved in patients with idiopathic PAH (IPAH) due to vasodilators uses, there is no such trend in patients with SSc-PAH [2].

The weighted gene coexpression network analysis (WGCNA), which has been widely used to explore gene networks' characteristics related to complex diseases [12], can be used to investigate the association between the genome and clinical features and identify candidate biomarkers. This study's objective was to use WGCNA analysis to investigate the gene-network characteristics of peripheral blood associated with SSc-PAH and to identify the hub genes most relevant to SSc-PAH and to use gene set enrichment analysis (GSEA) of a single candidate gene to explore their potential function.

## 2. Materials and Methods

### 2.1. Gene Expression Data and Data Preprocessing

The dataset of RNA expression profiles of SSc-PAH was driven from the Gene Expression Omnibus (GEO) database (https://www.NCBI.nlm.nih.gov/GEO/). In this study, the microarray dataset GSE33463 [13] from whole peripheral blood in 42 SSc-PAH patients and 41 controls was obtained to build coexpression networks and identify hub genes related to SSc-PAH. Considering that the datasets used in this study were downloaded from the GEO database through publicly available methods, the imperative of informed consent was waived.

The R software (version 3.5.2; https://www.R-project.org/) and several packages were used for data mining and statistical analysis. Meaningfully, the “normalizeBetweenArrays” function of the “Limma” package [14] was used for normalization.

### 2.2. Screening of Differentially Expressed Genes

We screened the differentially expressed genes (DEGs) between SSc-PAH patients and normal controls in the expressing data by using the *t*-test in the “Limma” package in R. The cut-off criteria were defined as ∣ log2 FC  | >0.5, and adjusted *P* values <0.05.

### 2.3. Construction of Coexpression Network

Through the R package “WGCNA” [12], the DEGs' coexpression network based on GSE33463 was constructed. In the coexpression network, based on the soft-thresholding power of 16 and the minimum number of genes in the module of 30, genes with high absolute correlations were clustered into the same module.

### 2.4. Gene Ontology and Pathway Enrichment Analysis

It would be more productive if gene ontology and pathway enrichment analysis were performed on a transcriptional module closely related to the trait. To further probe the function of the DEGs in the key module, the Gene Ontology (GO) analysis and the Kyoto Encyclopedia of Genes and Genomes (KEGG) pathway analysis were performed using the R package “clusterProfiler” [15], where GO analysis included molecular function (MF), biological processes (BP), and cellular component (CC). *P* < 0.05 was set as the cut-off standard.

### 2.5. Identification of Hub Genes in Clinically Significant Modules

Gene significance (GS) was evaluated by the mediated *P* value (GS = *lgP*) of each gene in a linear regression between gene expression and the clinical characteristics [16], while the genes' connectivity was assessed by the absolute value of Pearson's correlation.

In our study, genes with GS greater than 0.95 and ModuleMembership > 0.9 were considered to be the hub genes of the modules, reflecting a meaningful correlation with particular clinical features.

### 2.6. Genes Validation and Efficacy Evaluation

A second dataset GSE19617 [17], obtained from RNA extracted from peripheral blood mononuclear cells (PBMCs) in 17 patients with SSc-PAH and 12 healthy controls, was used to verify the hub genes. To evaluate the hub genes' reliability to distinguish SSc-PAH patients from healthy controls, we plotted receiver operating characteristic (ROC) curves and calculated the area under curves (AUCs).

### 2.7. Gene Set Enrichment Analysis

GSEA of a single candidate gene was conducted using the R package “clusterProfiler” to explore the potential function of the proper candidate genes in SSc-PAH. We use the h.all.v7.0.entrez.gmt in the Molecular Signatures Database as the reference gene set. We chose the adjusted *P* value <0.05 as the cut-off criterion.

## 3. Results

### 3.1. Differentially Expressed Genes between SSc-PAH and Normal Controls

A total of 938 DEGs were identified in the gene expression microarray study of 42 SSc-PAH patients and 41 controls, and the top 15 up-regulated genes and 15 down-regulated genes are shown in Table 1. These DEGs were selected for subsequent analysis.

### 3.2. Coexpression Network Construction of the SSc-PAH and Normal Conditions

**Figure 1:**
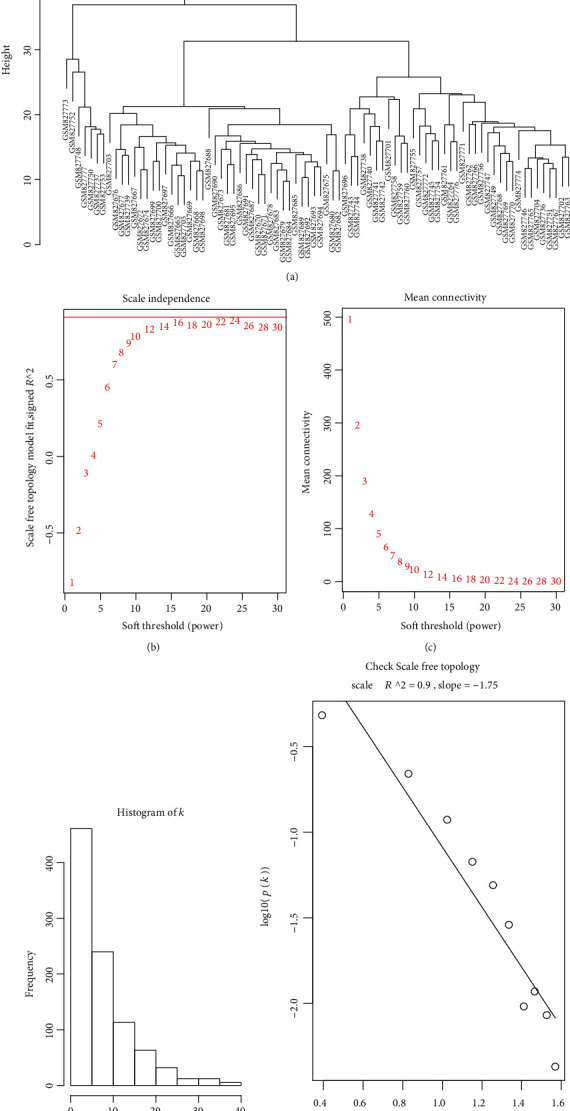
(a) Sample clustering to detect outliers. When the cut-off height was set at 40, one of the samples was found to be biased. (b) Analysis of the scale-free fit index for various soft-thresholding powers and (c) analysis of the mean connectivity for various soft-thresholding powers. (d, e) exhibited the reliability of scale-free topology when *β* = 16.

**Figure 2:**
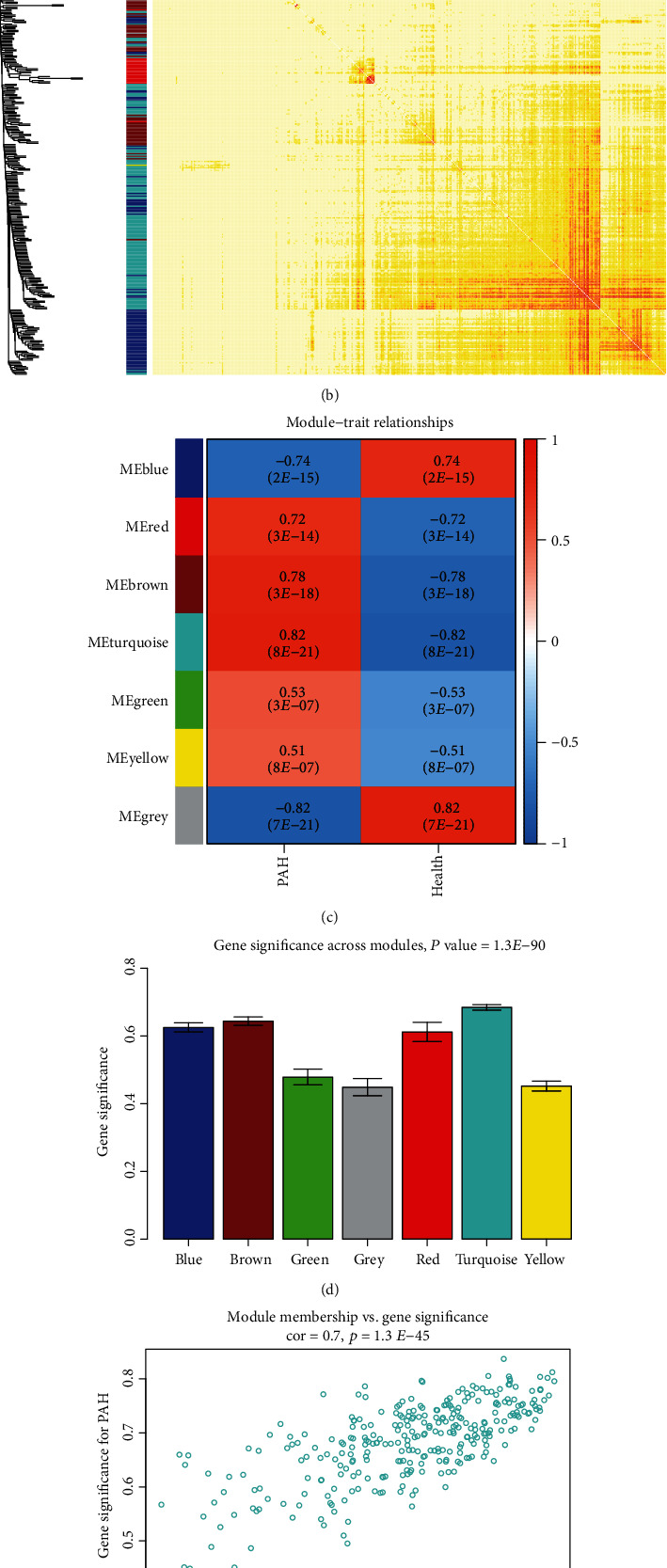
(a) Clustering dendrogram of differentially expressed genes associated with systemic sclerosis-related pulmonary arterial hypertension (SSc-PAH). (b) Network heat map in the coexpression module (as the color deepened, the overlap gradually increased). (c) Heat map of the correlation between module eigengenes and clinical traits of systemic sclerosis-related pulmonary arterial hypertension. (d) Distribution of average gene significance and errors in the modules related to systemic sclerosis-related pulmonary arterial hypertension. (e) The gene significance for systemic sclerosis-related pulmonary arterial hypertension in the turquoise module (one dot represents one gene in the turquoise module). (f) The top 16 genes with high gene significance for sclerosis-related pulmonary arterial hypertension were tightly related to each other. PAH: pulmonary arterial hypertension.

After using the average method of the “hclust” function to evaluate the expression matrix, the gene chip (GSM827775) with a cluster height exceeding 40 exhibited deviation and was excluded from further analysis (Figure 1(a)). The soft-thresholding parameter was selected as 16 (scale-free *R*^2^ = 0.9) to ensure a scale-free network when 0.9 was used as the correlation coefficient threshold (Figures 1(b) and 1(c)). Seven coexpression modules were constructed using WGCNA analysis (Figure 2(a)), which contained the most genes in was the turquoise module. And these coexpression modules were independent of other modules (Figure 2(b)).

### 3.3. Identification of Clinically Significant Modules and Hub Genes

Since the turquoise module had the highest correlation with SSc-PAH and a high correlation with clinical characteristics among all modules, the turquoise module was selected for further analysis (Figures 2(c) and 2(d)). Based on the cut-off criteria (∣MM | >0.95 and ∣GS | >0.90), 27 genes with high connectivity in the clinically important module were identified as hub genes. Notably, some genes in the turquoise module, including “BTG3,” “C12orf41,” “CCR2,” “COPB2,” “DYNLL2,” “ETNK1,” “GIMAP4,” “GIMAP8,” “HSPA1A,” “LFNG,” “LOC653171,” “LOC731878,” “RAB10,” “TMEM60,” “TNFAIP3,” and “TNFAIP8L2,” had high gene significance for SSc-PAH (Figures 2(e) and 2(f)). This indicated that these genes mentioned above were also closely correlated with each other (Figure 2(f)).

### 3.4. Functional Annotation of the Key Module

GO analysis showed that the genes in the turquoise module were mainly enriched in C-C chemokine receptor activity, C-C chemokine binding, G protein-coupled chemoattractant receptor activity, and chemokine receptor activity. The relationship between these genes and GO terms suggested that many genes were linked to immune response and inflammation (Table 2). No significant results were observed for KEGG enrichment.

### 3.5. Validation and Efficacy Evaluation of Hub Genes

**Figure 3:**
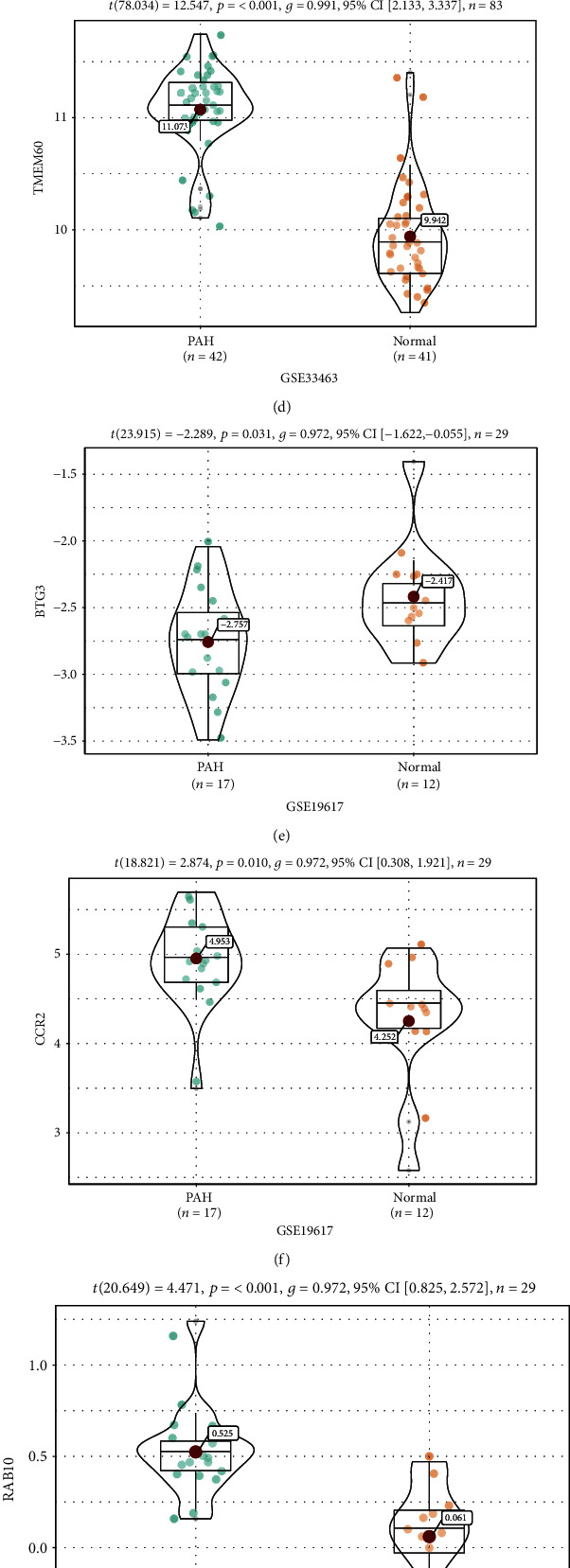
(a–h) Expression of hub genes. Expression levels of BTG3 (a), CCR2 (b), RAB10 (c), and TMEM60 (d) of dataset GSE33463. Expression levels of BTG3 (e), CCR2 (f), RAB10 (g), and TMEM60 (h) of dataset GSE19617. (i, j) Receiver operating characteristic curve of hub genes (BTG3, CCR2, RAB10, and TMEM60) in the two datasets GSE33463 (i) and GSE19617 (j). BTG3: B cell translocation gene 3; CCR2: C-C motif chemokine receptor 2; RAB10: member RAS oncogene family; TMEM60: transmembrane protein 60.

In dataset GSE33463, the expression of four genes, B cell translocation gene 3 (BTG3), C-C motif chemokine receptor 2 (CCR2), member RAS oncogene family (RAB10), and transmembrane protein 60 (TMEM60), was notably increased or decreased in the PBMCs of SSc-PAH patients compared with the controls (Figures 3(a)–3(d)). In addition, in the second dataset GSE19617, the expression of BTG3, CCR2, RAB10, and TMEM60 (all *P* < 0.05) was also considerably up-regulated or down-regulated in the SSc-PAH patients (Figures 3(e)–3(h)). Furthermore, to distinguish SSc-PAH from the controls, we used the ROC curves to calculate the AUCs. The AUC of each gene in datasets GSE19617 and GSE33463 was greater than 0.7 (Figures 3(i) and 3(j)).

### 3.6. Gene Set Enrichment Analysis

**Figure 4:**
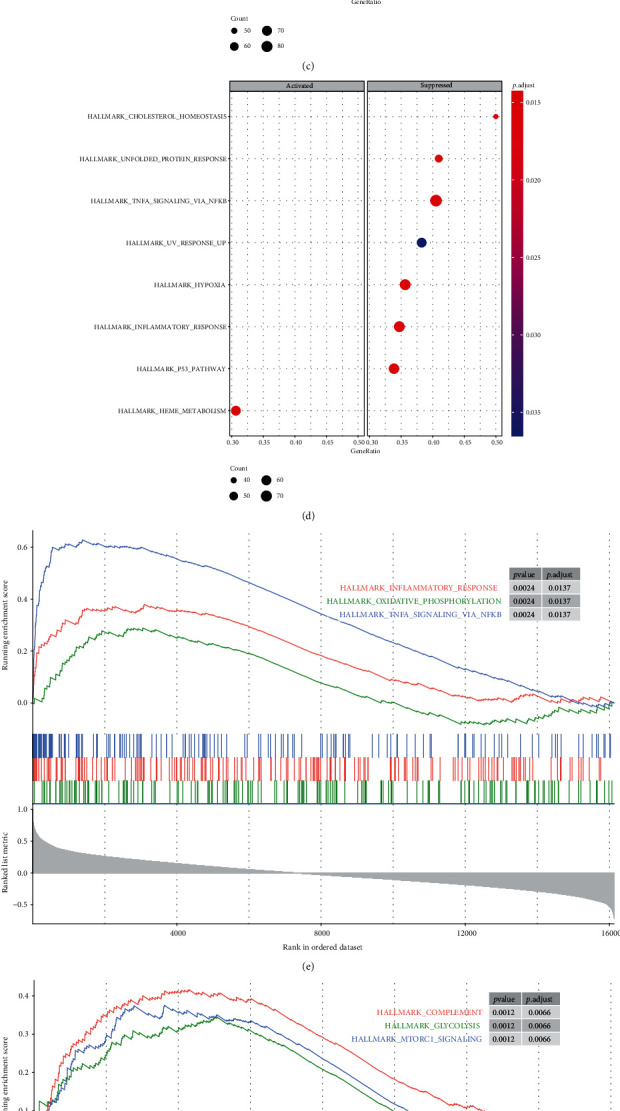
(a–d) Gene set enrichment analysis (GSEA). The full list of gene sets enriched in samples with a high expression of BTG3 (a), CCR2 (b), RAB10 (c), and TMEM60 (d). (e–g) Gene sets related to immunity and inflammation. Gene sets related to immunity and inflammation enriched in samples with a high expression of BTG3 (e), CCR2 (f), RAB10 (h), and TMEM60 (g). BTG3: B cell translocation gene 3; CCR2: C-C motif chemokine receptor 2; RAB10: member RAS oncogene family; TMEM60: transmembrane protein 60.

The complete list of gene sets enriched in samples with a high expression of BTG3, CCR2, RAB10, or TMEM60 was found through GSEA (Figures 4(a)–4(d)). The gene sets related to immunity and inflammation among the complete list were used for further analysis. “Complement” was enriched in samples in which CCR2 and RAB10 were highly expressed (Figures 4(f) and 4(g)). Similarly, the samples with a high expression of BTG3 and TMEM60 were enriched in “inflammatory response” (Figures 4(e) and 4(h)). Moreover, “tumor necrosis factor-*α* (TNF-*α*) signaling via nuclear factor-kappa B (NF-*κ*B)” and “mammalian target of rapamycin complex 1 (mTORC1) signaling” were enriched in the samples with a high expression of any one of BTG3, CCR2, RAB10, or TMEM60 (Figures 4(e)–4(h)).

## 4. Discussion

Recent studies have provided new clues into the critical signaling pathways of PAH. These signaling pathways include inflammation, immune activation, endothelial dysfunction, and growth factors [5]. In this study, we built a coexpression network of SSc-PAH-related genes through WGCNA analysis. The association of modules and traits was constructed and visualized as a heat map to find the modules most relevant to PAH. The turquoise module was the most important in SSc-PAH, and hub genes in this module associated with SSc-PAH pathogenesis were discovered. The study's outcome indicated that candidate genes identified in SSc-PAH were associated with immunity, inflammation, and cytokines.

Some genes deemed as hub genes could play significant roles in the pathogenesis of SSc-PAH. Pulmonary vascular cells in PAH have similar phenotypic characteristics to tumor cells in hyperproliferation and antiapoptosis [18]. By down-regulating BTG3, miR-142-5p promotes the proliferation of vascular smooth muscle cells [19]. Although some findings indicated that CCR2 could not directly promote PAH development, it might play a previously unrecognized role in developing and remodeling pulmonary blood vessels [20]. Guanosine-5′-triphosphatase IMAP family member 4 (GIMAP4) is a locus that strongly affects susceptibility to vasculitis [21]. Considering the anti-inflammatory properties of heat shock protein A1A (HSPA1A) [22], low levels of intracellular and circulating HSPA1A would promote the proinflammatory state and increase the vulnerability of the arterial wall to the destructive effects of vascular risk factors connected with endothelial dysfunction [23]. Previous studies have shown that decreased expression of tumor necrosis factor-*α*-induced protein 8 (TNFAIP8), leading to reduced levels of vascular endothelial growth factor receptor 2 (VEGFR-2) [24]. The antiapoptotic effects could partially mediate the therapeutic effects of endothelial progenitor cell transplantation in PAH. However, the antiapoptotic effect of the conditioned medium of endothelial progenitor cells was attenuated by blocking VEGFR-2 [25]. Similarly, Pendergrass et al. found inflammatory mediators such as TNF-*α* and markers of vascular injury such as VEGF in SSc-PAH subjects [17]. Grigoriev et al. confirmed by real-time PCR that VEGF was significantly up-regulated in mild cases compared with severe PAH and healthy controls [26]. Polymorphisms within the tumor necrosis factor-*α*-induced protein 3 (TNFAIP3) genomic locus have been linked to multiple inflammatory and autoimmune diseases [27]. These genes have not been further elucidated in SSc-PAH. Hemmes et al. successfully distinguished the vasodilator-responsive and nonresponsive forms of PAH, but there are few relevant studies on SSc-PAH [28].

Among hub genes, in dataset GSE33463, the expression of BTG3, CCR2, RAB10, and TMEM60 was significantly increased or decreased in the SSc-PAH patients compared with healthy controls. Simultaneously, we confirmed the above four hub genes' expression levels in GSE19617, and their expression in PBMCs was also significantly up- or down-regulated. In the future, more experiments are needed to illuminate their expression and related functions in different ethnic groups.

Because the genes within a module were closely related in function, we performed GO analysis to investigate genes' biological functions in the turquoise module. The results indicated that the genes were mainly enriched in C-C chemokine receptor activity, C-C chemokine binding, G protein-coupled chemoattractant receptor activity, and chemokine receptor activity. There was no doubt that SSc manifested itself as an immune system disorder and endothelial dysfunction [29]. Various studies have shown that the immune processes originating in the lungs seemed to promote PAH development and were likely to leave evident fingerprints in the systemic circulation [30]. Hemmes et al. found extensive differences in RNA expression patterns using microarray analysis, such as cell-cell adhesion factors [28].

Furthermore, our study revealed that “inflammatory response” and “complement” were involved in the pathogenesis of SSc-PAH. Immunoglobulin-driven complement activation could regulate proinflammatory remodeling in PAH [31]. Besides, “TNF-*α* signaling via NF-*κ*B” and “mTORC1 signaling” were also enriched. TNF-*α*, one of the inflammatory mediators, was found in SSc-PAH subjects [17]. Baicalein inhibited pulmonary artery remodeling in rats at least in part through NF-*κ*B pathways [32]. In mice, loss of the D prostanoid receptor subtype one aggravated vascular remodeling in PAH via mTORC1 signaling [33]. Aldosterone could upregulate the mammalian target of the rapamycin complex one subunit raptor, inducing abnormal survival patterns of pulmonary artery smooth muscle cells to promote PAH [34].

Our study also had limitations. First, due to GEO's incomplete data, some patients' characteristics were unknown, including autoantibodies. Second, many biomarkers related to SSc-PAH remain puzzling, and further bioinformatics analysis and experimental confirmation are needed to detail the biological functions of these genes in SSc-PAH. Third, we used data from only two different studies in our WGCNA analysis and validation of hub genes. Microarray samples need to be extracted from patients with varying degrees of PAH, and more samples are needed.

## 5. Conclusions

In summary, we identified the hub genes in the key gene coexpression modules, and the four genes (BTG3, CCR2, RAB10, and TMEM60) play an essential role in the diagnosis of SSc-PAH patients. Some functional biological pathways linking immunity, inflammation, and cytokines play a critical role in the pathogenesis of SSc-PAH. These results provide new insights into SSc-PAH diagnosis and treatment, although the precise mechanisms still require further exploration.

## Figures and Tables

**Table 1:** The top 15 up-regulated genes and 15 down-regulated genes identified in the microarray dataset GSE33463.

Gene symbol	logFC	*P* value	Adj. *P* value
MOAP1	1.051413956	1.25*E*-21	2.01*E*-17
MED10	0.757768783	1.77*E*-21	2.01*E*-17
EIF1	0.949347975	9.48*E*-21	4.91*E*-17
DNAJB1	1.235358669	9.76*E*-21	4.91*E*-17
MYLIP	1.145187023	1.42*E*-20	5.36*E*-17
DYNLL2	1.300670563	2.45*E*-20	7.69*E*-17
SBDS	1.160211817	5.46*E*-20	1.18*E*-16
CCDC59	0.943865194	5.61*E*-20	1.18*E*-16
PITPNC1	0.729721186	7.93*E*-20	1.53*E*-16
LOC652773	0.833258361	2.19*E*-19	3.07*E*-16
LOC388275	0.765982809	3.01*E*-19	3.79*E*-16
MGAT4A	1.340399036	6.79*E*-19	7.77*E*-16
TOMM20	0.627208790	7.46*E*-19	8.16*E*-16
TIGA1	0.820889344	8.78*E*-19	9.20*E*-16
IRF2BP2	0.974930681	1.42*E*-18	1.38*E*-15
TMEM60	-1.131294044	2.40*E*-21	2.01*E*-17
EXOSC3	-0.835661131	1.49*E*-20	5.36*E*-17
ASH2L	-0.588897110	3.02*E*-20	8.44*E*-17
GIMAP4	-1.639178038	4.24*E*-20	1.07*E*-16
FPR2	-1.495763022	1.10*E*-19	1.85*E*-16
TNFAIP8L2	-0.909455924	1.51*E*-19	2.37*E*-16
SCO1	-0.615800262	1.67*E*-19	2.48*E*-16
CRIPT	-0.737821571	2.59*E*-19	3.43*E*-16
NFE2	-1.595721981	5.07*E*-19	6.07*E*-16
RNF149	-1.090753168	9.18*E*-19	9.24*E*-16
COPB2	-0.823346942	1.97*E*-18	1.77*E*-15
APEX2	-0.546065608	2.34*E*-18	2.03*E*-15
SAMD9L	-1.095950962	2.65*E*-18	2.17*E*-15
LFNG	-1.479067449	2.84*E*-18	2.23*E*-15

**Table 2:** Gene ontology enrichment analysis of turquoise module genes.

Term	Description	Count	*P* adjust
GO:0016493	C-C chemokine receptor activity	4	0.029688
GO:0019957	C-C chemokine binding	4	0.029688
GO:0001637	G protein-coupled chemoattractant receptor activity	4	0.029688
GO:0004950	Chemokine receptor activity	4	0.029688
GO:0019956	Chemokine binding	4	0.045889
GO:0004519	Endonuclease activity	7	0.045889
GO:0004518	Nuclease activity	9	0.045889
GO:0005159	Insulin-like growth factor receptor binding	3	0.045889
GO:0061630	Ubiquitin protein ligase activity	9	0.045889
GO:0004521	Endoribonuclease activity	5	0.045889
GO:0061659	Ubiquitin-like protein ligase activity	9	0.048451

## Data Availability

The datasets analyzed during this study were publicly available databases, such as the Gene Expression Omnibus (GEO) dataset.
